# Human Botryomycosis

**Published:** 1910-12-03

**Authors:** 


### TROPICAL MEDICINE.

### HUMAN BOTRYOMYCOSIS.

At the British Medical Association meeting held in London last July Captain R. G. Archibald, Pathologist to the Wellcome Research Laboratories in Khartoum, read a paper on Human Botryomycosis, a new tropical malady. The condition, a tumour formation, was found on the scalp of a native from the Sudan, and when sections of this were made and suitably stained, the presence of agglomerations and collections of circular disc-like masses were noted, lying irregularly throughout the growth. They exhibited a close resemblance to staphylococci of various sizes, and their similarity to bunches of grapes prompted the name Botryomycosis (*βότρυς*= a grape; *μύκης*= fungus). In addition to the coccal masses, three of the seven tumours examined also exhibited a streptothrix closely resembling that met with in Madura foot disease. After the discovery of the first case, similar tumours were removed from other individuals from the breast, arms, hand, feet, and scrotum, and also from camels; the infection is thus a fairly widespread one.

The disease commences, as a rule, in the form of a superficial nodule, which increases in size and invades the deeper tissues, involving fasciæ, muscles, tendons, and sometimes the bones. The surface of the tumour eventually breaks down and the openings of many sinuses, from which a thin watery pus containing yellow-white granules exudes, then become visible.

Around these, masses of white fibrous tissue develop, the lumen being lined by granulation and necrosed tissues. A section made through one of these growths at once reveals their fibrotic nature, the intervening areas between the sinuses consisting for the most part of white fibrous tissue, frequently arranged in whorls, and of sharply demarcated yellow hyaline-looking areas, which microscopically are seen to be composed of groups of fat-cells. Captain Archibald believes that, from the fact of a streptothrix condition being present in three of the tumours, that botryomycosis is really a streptothricosis, and that the clumps of botryomyces represent the gonidia of possibly a new and hitherto undescribed species of streptothrix. Certainly there were no signs of the large amœba described by Letulle and others. Further investigations may settle the matter one way or the other and definitely decide what is the causative agent of this disease. Many of these chronic granulomatous conditions are now being run to earth, the finding of *Leishmania tropica* in lesions of the skin in Khartoum having cleared the way for further investigations and classifications of chronic skin lesions in Egypt and the tropics generally.

